# Supplementary material for: Medical ontology learning framework to investigate daytime impairment in insomnia disorder and treatment effects
Source: Commun Med (Lond). 2025 Feb 28;5:54. doi: 10.1038/s43856-024-00698-2 (PMC11871003; doi:10.1038/s43856-024-00698-2)
Supplement: Supplementary file 2 — Supplementary Material [file 43856_2024_698_MOESM2_ESM.docx]

**Supplementary Material**

**Supplementary Methods**

*Insomnia experts*

The following provides a description of the qualifications of the insomnia experts consulted for this project.

Insomnia expert 1

- Certified by the American Board of Psychiatry and Neurology in three separate boards: General Psychiatry, Geriatric Psychiatry, and Sleep Medicine
- Was an invited author of the first Board Exam in Sleep Medicine
- Was the Director of the National Sleep Medicine Course
- Was on the Board of Directors for the American Academy of Sleep Medicine
- Their research as a principal investigator in the topic of sleep disorders has been supported by the National Institutes of Health (NIH) and other Foundations.

Insomnia expert 2

- Board Certified in Behavioral Sleep Medicine by the American Board of Sleep Medicine
- Board Certified in Behavioral Sleep Medicine by the Board of Behavioral Sleep Medicine
- Elected Fellow of the American Academy of Sleep Medicine, American Heart Association, and Society of Behavioral Sleep Medicine
- Past President of the Society of Behavioral Sleep Medicine
- Over 250 academic publications on the topic of sleep health
- Their research on the topic of sleep disorders has been funded by multiple institutes at the National Institutes of Health, as well as the Department of Defense and other Foundations.

*Supplementary Table 1: International Classification of Diseases (ICD) codes used to identify patients diagnosed with insomnia*

| **Description** | **ICD-10 Code** |
| --- | --- |
| Insomnia | G47.0 |
| Insomnia unspecified | G47.00 |
| Insomnia due to medical condition | G47.01 |
| Other insomnia | G47.09 |
| Other sleep disorders | G47.8 |
| Sleep disorder, unspecified | G47.9 |
| Insomnia not due to a substance or known physiological condition | F51.0 |
| Primary insomnia | F51.01 |
| Adjustment insomnia | F51.02 |
| Paradoxical insomnia | F51.03 |
| Psychophysiologic insomnia | F51.04 |
| Insomnia due to other mental disorder | F51.05 |
| Other insomnia not due to a substance or known physiological condition | F51.09 |

*Supplementary Table 2: Final model parameters*

| **Hyperparameter** | **Value** |
| --- | --- |
| alpha | 0.05 |
| min_alpha | 0.0001 |
| epochs | 10 |
| sg | 1 |
| model_type | Word2vec |
| vector_size | 300 |
| window | 8 |

*Supplementary Table 3: Hyperparameter search space*

| **Hyperparameter** | **Optimized Range** |
| --- | --- |
| alpha | [0.001, 0.009] |
| min_alpha | [0.0001, 0.0009] |
| epochs | [5, 75] |
| sg | {0, 1} |
| model_type | {"word2vec", "fastText"} |
| vector_size | {128, 256, 300, 1200, 2400, 3200} |
| window | [4, 12] |

*Supplementary Table 4: Insomnia expert-informed representation baseline*

| **ICD-10 Code** | **Code Description** | **Domain** |
| --- | --- | --- |
| R53.82 | Chronic fatigue, unspecified | Cognition, Emotional, Physical |
| R53.83 | Other fatigue | Cognition, Emotional, Physical |
| R40.0 | Somnolence | Cognition, Physical |
| R41.0 | Disorientation, unspecified | Cognition |

*Footnote:ICD-10, International Classification of Diseases, 10th revision.*

Supplementary Table 5: Semantic type daytime impairment

| **Semantic supertype** | **Semantic type** |
| --- | --- |
| Daytime impairment | Activity |
| Daytime impairment | Finding |
| Daytime impairment | Functional Concept |
| Daytime impairment | Mental Process |
| Daytime impairment | Mental or Behavioral Dysfunction |
| Daytime impairment | Qualitative Concept |
| Daytime impairment | Quantitative Concept |
| Daytime impairment | Sign or Symptom |
| Daytime impairment | Individual Behavior |
| Daytime impairment | Disease or Syndrome |
| Daytime impairment | Organism Function |
| Daytime impairment | Daily or Recreational Activity |
| Daytime impairment | Social Behavior |

*We used the Unified Medical Language System (UMLS) Application Programming Interface (API) to assign types to each token. To compare daytime impairments, we defined a supertype for daytime impairments. This supertype consists of the UMLS semantic types listed above.*
