# Supplementary material for: Medical ontology learning framework to investigate daytime impairment in insomnia disorder and treatment effects
Source: Commun Med (Lond). 2025 Feb 28;5:54. doi: 10.1038/s43856-024-00698-2 (PMC11871003; doi:10.1038/s43856-024-00698-2)
Supplement: Supplementary file 3 — Description of Additional Supplementary Files [file 43856_2024_698_MOESM3_ESM.pdf]

# Description of Additional Supplementary Files

File name: Supplementary Data 1
Description: Model input also referred to as simple ontology

File name: Supplementary Data 2
Description: Daytime impairment ontology

File name: Supplementary Data 3
Description: Insomnia expert-informed representation using DiSMOL synonyms

File name: Supplementary Data 4
Description: Word pairs with known relationships

File name: Supplementary Data 5
Description: The source data for Figure 3
