# Supplementary material for: Medical ontology learning framework to investigate daytime impairment in insomnia disorder and treatment effects
Source: Commun Med (Lond). 2025 Feb 28;5:54. doi: 10.1038/s43856-024-00698-2 (PMC11871003; doi:10.1038/s43856-024-00698-2)
Supplement: Supplementary file 4 — Supplementary Data 1 [file 43856_2024_698_MOESM4_ESM.docx]

**Supplementary Data 1**

| **Domain** | **Item** | **Synonyms from insomnia experts** |
| --- | --- | --- |
| Cognition | clear-headed | attentive |
|  |  | oriented |
|  |  | foggy |
|  |  | disoriented |
|  |  | clear_head |
|  |  | alert |
|  |  | aware |
| Cognition | concentration | attention |
|  |  | focus |
|  |  | vigilance |
|  |  | concentrated |
| Cognition | forgetful | cognitive_disorder |
|  |  | amnesia |
|  |  | memory_impairment |
|  |  | inattentive |
|  |  | forgetfully |
| Emotional | worried | fearful |
|  |  | hypervigilant |
|  |  | anxiety |
|  |  | stress |
| Emotional | frustrated | discouraged |
|  |  | defeated |
|  |  | dissatisfied |
|  |  | annoyed |
| Emotional | irritable | annoyed |
|  |  | testy |
|  |  | crankiness |
| Emotional | stressed | burdened |
|  |  | overwhelmed |
|  |  | anxious |
|  |  | mood |
| Physical | energetic | vigor |
|  |  | stamina |
|  |  | alert |
|  |  | energy |
| Cognition | effort | depleted |
|  |  | burned_out |
|  |  | compensation |
|  |  | tired |
| Cognition | refreshed | invigorated |
|  |  | vigorous |
|  |  | ready |
|  |  | rested |
| Physical | mentally tired | depleted |
|  |  | burned_out |
|  |  | depressed |
|  |  | amotivation |
| Physical | physically tired | exhausted |
| Physical | sleepy | listless |
|  |  | sluggish |
|  |  | lethargic |
|  |  | fatigue |
|  |  | remain_awake |
| Cognition | awake | vigilant |
|  |  | aware |
