# Supplementary material for: Medical ontology learning framework to investigate daytime impairment in insomnia disorder and treatment effects
Source: Commun Med (Lond). 2025 Feb 28;5:54. doi: 10.1038/s43856-024-00698-2 (PMC11871003; doi:10.1038/s43856-024-00698-2)
Supplement: Supplementary file 5 — Supplementary Data 2 [file 43856_2024_698_MOESM5_ESM.docx]

**Supplementary Data 2**

| **Domain** | **Item** | **DiSMOL synonym** | **Semantic negation required** |
| --- | --- | --- | --- |
| Cognition | Clear-Headed | awake | Yes |
| Cognition | Clear-Headed | oriented | Yes |
| Cognition | Clear-Headed | understand | Yes |
| Cognition | Clear-Headed | concentrate | Yes |
| Cognition | Clear-Headed | mind | Yes |
| Cognition | Clear-Headed | sleepy | No |
| Cognition | Clear-Headed | dizzy | No |
| Cognition | Clear-Headed | remember | Yes |
| Cognition | Clear-Headed | confusion | No |
| Cognition | Clear-Headed | insight | Yes |
| Cognition | Clear-Headed | coherent | Yes |
| Cognition | Clear-Headed | clear | Yes |
| Cognition | Concentration | hyperactivity | No |
| Cognition | Concentration | concentrate | Yes |
| Cognition | Concentration | focus | Yes |
| Cognition | Concentration | concentration | Yes |
| Cognition | Concentration | attention | Yes |
| Cognition | Concentration | ADHD | No |
| Cognition | Concentration | asleep | No |
| Cognition | Concentration | sleep | No |
| Cognition | Forgetful | hyperactivity | No |
| Cognition | Forgetful | confusion | No |
| Cognition | Forgetful | knowledge | Yes |
| Cognition | Forgetful | forget | No |
| Cognition | Forgetful | ADHD | No |
| Cognition | Forgetful | memory | Yes |
| Cognition | Forgetful | dementia | No |
| Cognition | Forgetful | remember | Yes |
| Cognition | Forgetful | forgetful | No |
| Cognition | Effort | fatigued | No |
| Cognition | Effort | dizzy | No |
| Cognition | Effort | energy | Yes |
| Cognition | Effort | weak | No |
| Cognition | Effort | effort | No |
| Cognition | Refreshed | sleepy | No |
| Cognition | Refreshed | sleepiness | No |
| Cognition | Refreshed | somnolence | No |
| Cognition | Refreshed | tired | No |
| Cognition | Refreshed | fatigued | No |
| Cognition | Refreshed | hypersomnia | No |
| Cognition | Refreshed | tiredness | No |
| Cognition | Refreshed | narcolepsy | No |
| Cognition | Refreshed | relieve | No |
| Cognition | Refreshed | refreshed | Yes |
| Cognition | Awake | alert | Yes |
| Cognition | Awake | oriented | Yes |
| Cognition | Awake | awake | Yes |
| Emotional | Worried | panic | No |
| Emotional | Worried | stressed | No |
| Emotional | Worried | cry | No |
| Emotional | Worried | worried | No |
| Emotional | Worried | PTSD | No |
| Emotional | Worried | anxious | No |
| Emotional | Worried | worry | No |
| Emotional | Worried | nervousness | No |
| Emotional | Worried | stress | No |
| Emotional | Worried | anxiety | No |
| Emotional | Worried | emotional | No |
| Emotional | Frustrated | cry | No |
| Emotional | Frustrated | depressed | No |
| Emotional | Frustrated | frustrated | No |
| Emotional | Frustrated | depression | No |
| Emotional | Irritable | tearful | No |
| Emotional | Irritable | cry | No |
| Emotional | Irritable | stressed | No |
| Emotional | Irritable | irritable | No |
| Emotional | Stressed | stressed | No |
| Emotional | Stressed | tearful | No |
| Emotional | Stressed | anxious | No |
| Emotional | Stressed | worry | No |
| Emotional | Stressed | stress | No |
| Emotional | Stressed | panic | No |
| Emotional | Stressed | nervous | No |
| Emotional | Stressed | worried | No |
| Emotional | Stressed | anxiety | No |
| Emotional | Stressed | fatigued | No |
| Emotional | Stressed | emotional | No |
| Emotional | Stressed | nervousness | No |
| Physical | Energetic | awake | Yes |
| Physical | Energetic | oriented | Yes |
| Physical | Energetic | sleepy | No |
| Physical | Energetic | nap | No |
| Physical | Energetic | fatigued | No |
| Physical | Energetic | libido | No |
| Physical | Energetic | tired | No |
| Physical | Energetic | energy | Yes |
| Physical | Energetic | sleepiness | No |
| Physical | Energetic | energetic | Yes |
| Physical | Mentally Tired | depressed | No |
| Physical | Mentally Tired | sad | No |
| Physical | Mentally Tired | depression | No |
| Physical | Mentally Tired | tired | No |
| Physical | Physically Tired | nap | No |
| Physical | Physically Tired | tired | No |
| Physical | Sleepy | nap | No |
| Physical | Sleepy | asleep | No |
| Physical | Sleepy | sleep | No |
| Physical | Sleepy | tiredness | No |
| Physical | Sleepy | tired | No |
| Physical | Sleepy | sleepiness | No |
| Physical | Sleepy | drive | Yes |
| Physical | Sleepy | fatigued | No |
| Physical | Sleepy | dizzy | No |
| Physical | Sleepy | chill | No |
| Physical | Sleepy | somnolence | No |
| Physical | Sleepy | manic | Yes |
| Physical | Sleepy | drowsiness | No |
| Physical | Sleepy | weak | No |
| Physical | Sleepy | sleepy | No |
