# Supplementary material for: Medical ontology learning framework to investigate daytime impairment in insomnia disorder and treatment effects
Source: Commun Med (Lond). 2025 Feb 28;5:54. doi: 10.1038/s43856-024-00698-2 (PMC11871003; doi:10.1038/s43856-024-00698-2)
Supplement: Supplementary file 6 — Supplementary Data 3 [file 43856_2024_698_MOESM6_ESM.docx]

**Supplementary Data 3**

| **Domain** | **Item** | **DiSMOL Synonym** | **ICD-10 code** |
| --- | --- | --- | --- |
| Cognition | Clear-Headed | alert / alertness | none |
| Cognition | Clear-Headed | clear headed | none |
| Cognition | Clear-Headed | understand | none |
| Cognition | Clear-Headed | concentrate | R41.84 |
| Cognition | Clear-Headed | sleepy | G47.3, R40.0 |
| Cognition | Clear-Headed | dizzy | R42* |
| Cognition | Clear-Headed | remember | none |
| Cognition | Clear-Headed | insight | none |
| Cognition | Clear-Headed | coherent | none |
| Cognition | Clear-Headed | clear | none |
| Cognition | Concentration | attention / (neg) inattention / (neg) inattentive | R41.84, F90* |
| Cognition | Concentration | concentrate / concentration | R41.84 |
| Cognition | Concentration | focus / focused / focusing | none |
| Cognition | Concentration | (neg) hyperactivity | F90*, R41.84 |
| Cognition | Concentration | ADHD | F90* |
| Cognition | Forgetful | forgetful | F01*, F02*, F03*, F04*, G30*, G31.0*, R41.1, R41.2, R41.3 |
| Cognition | Forgetful | dementia | F01*, F02*, F03*, F04*, G30*, G31.0* |
| Cognition | Effort | effort | none |
| Cognition | Effort | (neg) fatigued | R53* |
| Cognition | Effort | energy | none |
| Cognition | Effort | weak | M62.81, R53.1 |
| Cognition | Refreshed | refreshed | none |
| Cognition | Refreshed | somnolence | R40.0, R40* |
| Cognition | Refreshed | tired | R53* |
| Cognition | Refreshed | fatigued | R53* |
| Cognition | Refreshed | hypersomnia | G47.1* |
| Cognition | Refreshed | narcolepsy | G47.41 |
| Cognition | Awake | awake | none |
| Cognition | Awake | alert | none |
| Cognition | Awake | awake, awoke, awaken, awakening | none |
| Emotional | Worried | worried | R45.82, Z71.1 |
| Emotional | Worried | anxiety / anxious | F41* |
| Emotional | Worried | general anxiety disorder (GAD) | F41.1 |
| Emotional | Worried | nervous | R45.0 |
| Emotional | Worried | PTSD | F43.1 |
| Emotional | Worried | emotional | R45.7 |
| Emotional | Frustrated | cry | R45.2 |
| Emotional | Frustrated | (neg) depressed | F20.4, F32*, F33*, F34.1, F41.2, F43.2, F53.0, F31.3, F31.4, F31.5, F31.75, F31.76, F31.81, F31.9, F41.8 |
| Emotional | Irritable | irritable | R45.4 |
| Emotional | Irritable | stress / stressed | F43*, Z73.3, Z63.7, R45.7, Z56.3 |
| Emotional | Stressed | stress / stressed / stressful | Z73.3, Z63.7, R45.7, Z56.3 |
| Emotional | Stressed | emotional | R45.7 |
| Emotional | Stressed | anxious | F40*, F41*, F42*, F43*, F44*, F45*, F48*, F60.6 |
| Emotional | Stressed | worried | R45.82, Z71.1 |
| Physical | Energetic | energetic / energized | none |
| Physical | Energetic | energy | none |
| Physical | Energetic | (neg) sleepy | R40.0 |
| Physical | Energetic | fatigued | R53* |
| Physical | Energetic | nap | none |
| Physical | Energetic | tired | R53* |
| Physical | Mentally Tired | mentally tired | R53.1, R53.8, R53.81, R53.82, R53.83 |
| Physical | Mentally Tired | tired, tiredness | R53* |
| Physical | Physically Tired | physically tired | R53*, T73.3* |
| Physical | Physically Tired | tired, tiredness | R53* |
| Physical | Physically Tired | nap | none |
| Physical | Sleepy | drowsily, drowsy, drowsiness | R40.0 |
| Physical | Sleepy | sleepy, sleepily | R40.0 |
| Physical | Sleepy | somnolence | R40.0 |
| Physical | Sleepy | tired, tiredly, tiredness | R53* |
| Physical | Sleepy | nap | none |
| Physical | Sleepy | sleepiness | G47.3 |
| Physical | Sleepy | drive | none |
| Physical | Sleepy | manic | F30.9 |
