# Supplementary material for: Medical ontology learning framework to investigate daytime impairment in insomnia disorder and treatment effects
Source: Commun Med (Lond). 2025 Feb 28;5:54. doi: 10.1038/s43856-024-00698-2 (PMC11871003; doi:10.1038/s43856-024-00698-2)
Supplement: Supplementary file 7 — Supplementary Data 4 [file 43856_2024_698_MOESM7_ESM.docx]

**Supplementary Data 4**

| **Token a** | **Token b** |
| --- | --- |
| attentive | oriented |
| attentive | foggy |
| attentive | disoriented |
| attentive | clear_head |
| attentive | clear_headed |
| attentive | alert |
| attentive | aware |
| oriented | foggy |
| oriented | disoriented |
| oriented | clear_head |
| oriented | clear_headed |
| oriented | alert |
| oriented | aware |
| foggy | disoriented |
| foggy | clear_head |
| foggy | clear_headed |
| foggy | alert |
| foggy | aware |
| disoriented | clear_head |
| disoriented | clear_headed |
| disoriented | alert |
| disoriented | aware |
| clear_head | clear_headed |
| clear_head | alert |
| clear_head | aware |
| clear_headed | alert |
| clear_headed | aware |
| alert | aware |
| attention | focus |
| attention | vigilance |
| attention | concentration |
| attention | concentrated |
| focus | vigilance |
| focus | concentration |
| focus | concentrated |
| vigilance | concentration |
| vigilance | concentrated |
| concentration | concentrated |
| cognitive_disorder | amnesia |
| cognitive_disorder | memory_impairment |
| cognitive_disorder | inattentive |
| cognitive_disorder | forgetful |
| cognitive_disorder | forgetfully |
| amnesia | memory_impairment |
| amnesia | inattentive |
| amnesia | forgetful |
| amnesia | forgetfully |
| memory_impairment | inattentive |
| memory_impairment | forgetful |
| memory_impairment | forgetfully |
| inattentive | forgetful |
| inattentive | forgetfully |
| forgetful | forgetfully |
| fearful | hypervigilant |
| fearful | anxiety |
| fearful | stress |
| fearful | worried |
| hypervigilant | anxiety |
| hypervigilant | stress |
| hypervigilant | worried |
| anxiety | stress |
| anxiety | worried |
| stress | worried |
| discouraged | defeated |
| discouraged | dissatisfied |
| discouraged | annoyed |
| discouraged | frustrated |
| defeated | dissatisfied |
| defeated | annoyed |
| defeated | frustrated |
| dissatisfied | annoyed |
| dissatisfied | frustrated |
| annoyed | frustrated |
| annoyed | testy |
| annoyed | crankiness |
| annoyed | irritable |
| testy | crankiness |
| testy | irritable |
| crankiness | irritable |
| burdened | overwhelmed |
| burdened | anxious |
| burdened | mood |
| burdened | stressed |
| overwhelmed | anxious |
| overwhelmed | mood |
| overwhelmed | stressed |
| anxious | mood |
| anxious | stressed |
| mood | stressed |
| vigor | stamina |
| vigor | alert |
| vigor | energy |
| vigor | energetic |
| stamina | alert |
| stamina | energy |
| stamina | energetic |
| alert | energy |
| alert | energetic |
| energy | energetic |
| depleted | burned_out |
| depleted | compensation |
| depleted | tired |
| depleted | effort |
| burned_out | compensation |
| burned_out | tired |
| burned_out | effort |
| compensation | tired |
| compensation | effort |
| tired | effort |
| invigorated | vigorous |
| invigorated | ready |
| invigorated | refreshed |
| invigorated | rested |
| vigorous | ready |
| vigorous | refreshed |
| vigorous | rested |
| ready | refreshed |
| ready | rested |
| refreshed | rested |
| depleted | burned_out |
| depleted | depressed |
| depleted | amotivation |
| depleted | mentally_tired |
| burned_out | depressed |
| burned_out | amotivation |
| burned_out | mentally_tired |
| depressed | amotivation |
| depressed | mentally_tired |
| amotivation | mentally_tired |
| exhausted | physically_tired |
| listless | sluggish |
| listless | lethargic |
| listless | fatigue |
| listless | remain_awake |
| listless | sleepy |
| sluggish | lethargic |
| sluggish | fatigue |
| sluggish | remain_awake |
| sluggish | sleepy |
| lethargic | fatigue |
| lethargic | remain_awake |
| lethargic | sleepy |
| fatigue | remain_awake |
| fatigue | sleepy |
| remain_awake | sleepy |
| vigilant | aware |
| vigilant | awake |
| aware | awake |
