# Supplementary material for: Medical ontology learning framework to investigate daytime impairment in insomnia disorder and treatment effects
Source: Commun Med (Lond). 2025 Feb 28;5:54. doi: 10.1038/s43856-024-00698-2 (PMC11871003; doi:10.1038/s43856-024-00698-2)
Supplement: Supplementary file 9 — Reporting Summary [file 43856_2024_698_MOESM9_ESM.pdf]

nature portfolio

Corresponding author(s): Alexander Büsser

Last updated by author(s): 18/9/23

Reporting Summary

Nature Portfolio wishes to improve the reproducibility of the work that we publish. This form provides structure for consistency and transparency in reporting. For further information on Nature Portfolio policies, see our Editorial Policies and the Editorial Policy Checklist.

Statistics

For all statistical analyses, confirm that the following items are present in the figure legend, table legend, main text, or Methods section.

n/a	Confirmed	
☐	☒	The exact sample size (*n*) for each experimental group/condition, given as a discrete number and unit of measurement
☐	☒	A statement on whether measurements were taken from distinct samples or whether the same sample was measured repeatedly
☐	☒	The statistical test(s) used AND whether they are one- or two-sided<br>*Only common tests should be described solely by name; describe more complex techniques in the Methods section.*
☐	☒	A description of all covariates tested
☐	☒	A description of any assumptions or corrections, such as tests of normality and adjustment for multiple comparisons
☐	☒	A full description of the statistical parameters including central tendency (e.g. means) or other basic estimates (e.g. regression coefficient) AND variation (e.g. standard deviation) or associated estimates of uncertainty (e.g. confidence intervals)
☐	☒	For null hypothesis testing, the test statistic (e.g. *F*, *t*, *r*) with confidence intervals, effect sizes, degrees of freedom and *P* value noted<br>*Give P values as exact values whenever suitable.*
☒	☐	For Bayesian analysis, information on the choice of priors and Markov chain Monte Carlo settings
☒	☐	For hierarchical and complex designs, identification of the appropriate level for tests and full reporting of outcomes
☐	☒	Estimates of effect sizes (e.g. Cohen's *d*, Pearson's *r*), indicating how they were calculated

Our web collection on statistics for biologists contains articles on many of the points above.

Software and code

Policy information about availability of computer code

Data collection	Due to the complex nature of Real World Data, various software packages for GP offices and hospital data systems were employed in multiple iterations. The data was procured through HealthVerity, a US-based data broker. We obtained licenses from two sources: one comprising closed medical claims, where the source provider remains undisclosed, and the other involving chart data from Amazing Charts LLC.
Data analysis	Data were analyzed using a suite of open access software packages, below is the list of the software packages used: Python==3.8, Pandas, Kedro==0.16.6, Seaborn==0.11, Scikit-Learn==0.22.2, Pyspark==3.2.1, numpy==1.18.1, optuna==2.10.1

For manuscripts utilizing custom algorithms or software that are central to the research but not yet described in published literature, software must be made available to editors and reviewers. We strongly encourage code deposition in a community repository (e.g. GitHub). See the Nature Portfolio guidelines for submitting code & software for further information.

Data

Policy information about availability of data

All manuscripts must include a data availability statement. This statement should provide the following information, where applicable:

- Accession codes, unique identifiers, or web links for publicly available datasets
- A description of any restrictions on data availability
- For clinical datasets or third party data, please ensure that the statement adheres to our policy

Restrictions apply to the general availability of the data because of patient agreements and the nature of patient data. Data was used under license for the study

presented in this manuscript.

Research involving human participants, their data, or biological material

Policy information about studies with human participants or human data. See also policy information about sex, gender (identity/presentation), and sexual orientation and race, ethnicity and racism.

Reporting on sex and gender	The data acquired via HealthVerity pertains exclusively to sex and not to gender. Sex was used to stratify the training and test set for the disease specific model development. In the study design to investigate the treatment effects of benzodiazepine, non-benzodiazepine and trazodone, factors related to sex were not taken into account, including matters like exclusion or inclusion criteria. This approach aligns with the fundamental goal of utilizing real-world data, which is to depict occurrences as they manifest in actual clinical practice.
Reporting on race, ethnicity, or other socially relevant groupings	Race, ethnicity was not measured in this real-world study.
Population characteristics	The study examined the population characteristics of three different groups: those prescribed Trazodone, non-benzodiazepine receptor agonists, and benzodiazepines. The mean age of participants in these groups varied slightly, with the benzodiazepine group being the oldest. The distribution of sex was similar across all three groups, with a majority of participants being female. The prevalence of certain conditions, such as arterial hypertension, diabetes Type II, anxiety, depression, psychiatric comorbidities, obesity, heart failure, ischemic heart disease, chronic obstructive pulmonary disease, and cerebral infarction, was examined across the three groups. More detailed information can be found in supplementary Table 9
Recruitment	Since the research constitutes a retrospective analysis of existing data and since it is based on real-world data, there was no specific recruitment process involved.
Ethics oversight	The study was approved by Idorsia's internal protocol review committee and the HealthVerity data utilization committee. The study was exempt from the requirement to obtain informed consent owing to the retrospective design.

Note that full information on the approval of the study protocol must also be provided in the manuscript.

Field-specific reporting

Please select the one below that is the best fit for your research. If you are not sure, read the appropriate sections before making your selection.

☒ Life sciences ☐ Behavioural & social sciences ☐ Ecological, evolutionary & environmental sciences

For a reference copy of the document with all sections, see nature.com/documents/nr-reporting-summary-flat.pdf

Life sciences study design

All studies must disclose on these points even when the disclosure is negative.

Sample size	Three distinct patient populations were identified based on the treatment administered: benzodiazepine (estazolam, flurazepam, lorazepam, quazepam, temazepam, triazolam) (n=1,045), non-benzodiazepine receptor agonists (eszopiclone, zaleplon, zolpidem) (n=2,361), and trazodone (n=1,522).
Data exclusions	All included patients were diagnosed with insomnia within 6 months prior to the start of treatment and were 18 years of age or older at the beginning of the baseline period. Patients were excluded if their most recent insomnia diagnosis date was greater than 6 months before treatment start, if they had received palliative care, had any reported active malignancies, or were pregnant. Observed data for all patients must be present for at least 365 days.
Replication	The HealthVerity dataset was split into a training and testing dataset. To increase reproducibility of the retrieved terms, eight models, trained on different bootstrap samples each using the best performing hyper-parameter configuration, were used to create an ensemble model. This involved averaging distances between tokens across the eight models to reduce variations in close neighbors due to small changes in the data and initialize the weights of the model. All disease representations DiSMOL, ICD-clin and ICD-clin-DiSMOL were tested on an independent test dataset. Replication of DiSMOL, ICD-clin and ICD-clin-DiSMOL, on an datset other than HealthVerity is left for future work.
Randomization	Selection of training and testing set for the development of the disease specific model was done by stratifying for age and sex. No retrospective adjustment was made to either set.
Blinding	The HealthVerity test set was kept blinded from the mathematicians and computer scientists until the development of DiSMOL was finalized. This test set was utilized solely for the algorithm's inference. Subsequently, the inferred results were compared against the ground truth determined by the sleep experts.

Reporting for specific materials, systems and methods

We require information from authors about some types of materials, experimental systems and methods used in many studies. Here, indicate whether each material, system or method listed is relevant to your study. If you are not sure if a list item applies to your research, read the appropriate section before selecting a response.

Materials & experimental systems

n/a	Involved in the study
☒	☐ Antibodies
☒	☐ Eukaryotic cell lines
☒	☐ Palaeontology and archaeology
☒	☐ Animals and other organisms
☒	☐ Clinical data
☒	☐ Dual use research of concern
☒	☐ Plants

Methods

n/a	Involved in the study
☒	☐ ChIP-seq
☒	☐ Flow cytometry
☒	☐ MRI-based neuroimaging
